# Overexpression of Circular *PRMT1* Transcripts in Colorectal Adenocarcinoma Predicts Recurrence and Poor Overall Survival

**DOI:** 10.3390/ijms26146683

**Published:** 2025-07-11

**Authors:** Panagiotis Kokoropoulos, Spyridon Christodoulou, Panagiotis Tsiakanikas, Panteleimon Vassiliu, Christos K. Kontos, Nikolaos Arkadopoulos

**Affiliations:** 1 Fourth Department of Surgery, University General Hospital “Attikon”, National and Kapodistrian University of Athens, 12462 Athens, Greece; kokoropoulos@yahoo.gr (P.K.); spyridon.christodoulou@yahoo.gr (S.C.); pant.greek@gmail.com (P.V.); narkado@hotmail.com (N.A.); 2 Department of Biochemistry and Molecular Biology, Faculty of Biology, National and Kapodistrian University of Athens, Panepistimiopolis, 15771 Athens, Greece; ptsiak@biol.uoa.gr

**Keywords:** colon cancer, circular RNA (circRNA), molecular tumor markers, prognosis, prognostic biomarkers, non-coding RNA

## Abstract

Colorectal cancer (CRC) is one of the most prevalent and deadly neoplasms globally; this fact puts emphasis on the need for accurate molecular biomarkers for early detection and accurate prognosis. Circular RNAs (circRNAs) have recently emerged as very promising cancer biomarkers. In this study, we thoroughly examined whether the expression levels of circular transcripts of the protein arginine methyltransferase 1 (*PRMT1*) gene can predict the prognosis of patients diagnosed with colorectal adenocarcinoma, the most frequent type of CRC. Hence, a highly sensitive quantitative PCR (qPCR) assay was developed and applied to quantify circ-PRMT1 expression in cDNAs from 210 primary colorectal adenocarcinoma tissue specimens and 86 paired normal colorectal mucosae. Extensive biostatistical analysis was then performed to assess the potential prognostic power of circ-PRMT1. Significant overexpression of this molecule was observed in colorectal adenocarcinoma tissue samples in contrast to their non-cancerous counterparts. Moreover, higher circ-PRMT1 expression was correlated with poorer disease-free survival (DFS) and worse overall survival (OS) in colorectal adenocarcinoma patients. Interestingly, multivariate Cox regression analysis revealed that the prognostic value of the expression of this circRNA does not depend on other established prognostic factors included in the prognostic model. Furthermore, the stratification of patients based on TNM staging revealed that higher circ-PRMT1 levels were significantly related to shorter DFS and OS intervals, particularly in patients with colorectal adenocarcinoma of TNM stage II or III. In summary, this original research study provides evidence that circ-PRMT1 overexpression represents a promising molecular biomarker of poor prognosis in colorectal adenocarcinoma, not depending on other established prognostic factors such as TNM staging.

## 1. Introduction

Colorectal cancer (CRC) develops predominately in glandular epithelial cells of the colon or rectum and is one of the most prevalent and deadly neoplasms globally, with 1.9 million new cases and 900,000 deaths reported in 2022. Incidence rates are highest in regions with high human development indexes and are rising worldwide, projected to reach 3.2 million annual cases by 2040. Primary risk factors for sporadic CRC include age (>50), obesity, tobacco use, alcohol consumption, and diets rich in pro-inflammatory foods. Nevertheless, ~30% of cases are linked to familial predisposition or hereditary syndromes [1]. CRC pathogenesis follows a stepwise progression from normal epithelium to adenoma, carcinoma, and ultimately metastatic disease. This transition is driven by accumulating genetic/epigenetic aberrations via three key molecular pathways: (a) chromosomal instability (CIN), marked by mutations in *APC*, *KRAS*, *PIK3CA*, and *TP53*; (b) the CpG island methylator phenotype (CIMP), characterized by widespread promoter hypermethylation and frequent *BRAF* mutations; and (c) microsatellite instability (MSI), linked to defective DNA mismatch repair (MMR) [1,2].

Because CRC often develops over decades, endoscopic polypectomy and stool-based screening tests are highly effective in the prevention and early detection of the disease. However, approximately 20% of patients present with metastatic disease at diagnosis and up to 50% of those diagnosed initially with localized tumors eventually relapse with distant metastases. Localized CRC is treated with curative resection of the primary tumor and regional lymphadenectomy. Adjuvant oxaliplatin–fluoropyrimidine chemotherapy is recommended for stage III and high-risk stage II colon cancers to prevent recurrence. In contrast, locally advanced rectal cancers (stages II–III) typically receive neoadjuvant chemoradiotherapy, followed by total mesorectal excision [3,4]. For metastatic CRC, first-line treatment usually combines fluoropyrimidines with oxaliplatin or irinotecan. The efficacy of cytotoxic agents is further enhanced when combined with targeted therapies such as anti-VEGF monoclonal antibodies (e.g., bevacizumab) or anti-EGFR inhibitors (e.g., cetuximab, panitumumab), the latter used exclusively for *RAS* wild-type tumors. Additionally, tumors with MSI-high/mismatch repair deficiency (dMMR) respond to immune checkpoint inhibitors like pembrolizumab, underscoring the role of biomarker-driven precision therapy in CRC [5].

Prognosis is predominantly determined by pathological TNM staging; however, molecular profiling is increasingly integrated into the personalized management of CRC [6]. Standard molecular biomarkers, including MMR/MSI status and KRAS/NRAS/BRAF mutations, provide both prognostic and predictive value [7]. Emerging tools such as the consensus molecular subtype (CMS) classification and established multigene panels further refine the risk stratification of CRC patients [8]. Similarly, novel biomarkers like circulating tumor DNA (ctDNA) are under investigation for the early detection and monitoring of therapeutic response during post-treatment surveillance [9]. Collectively, these advances are shifting CRC care away from a “one-size-fits-all” paradigm toward interventions tailored to individual tumor biology.

The post-transcriptional processing of eukaryotic pre-mRNAs includes splicing, 5′ capping, polyadenylation, and RNA editing. Alternative splicing (AS) is a critical regulatory step between transcription and translation that ultimately enhances both transcriptomic and proteomic diversity. In CRC, dysregulated AS patterns are associated with oncogenic phenotypes, including sustained proliferation, apoptosis evasion, metabolic reprogramming, drug resistance, and metastasis [10,11]. Back-splicing is a unique form of AS that generates single-stranded, circular RNAs (circRNAs) by covalently linking a downstream splice donor site to an upstream splice acceptor site, forming a closed RNA loop lacking 5′ cap and a 3′ poly (A) tail. Compared to canonical splicing, back-splicing is less efficient and depends on *cis*-acting RNA motifs such as intronic complementary sequences, as well as *trans*-acting factors that stabilize these sequences to enable circularization [12].

circRNAs are characterized by increased cytoplasmic stability due to their exonuclease-resistant closed conformation and are found frequently dysregulated in CRC. They facilitate tumor progression by regulating gene expression through miRNA sequestration, RNA-binding protein interactions, or unique peptide translation [12]. For example, circCAMSAP1 is implicated in tumor progression by sponging miR-328-5p, a tumor-suppressor miRNA, which typically represses the oncogenic transcription factor *E2F1* [13]. Aberrant circRNA expression disrupts cell cycle regulators and activates oncogenic pathways. Furthermore, circRNAs promote metastasis by inducing epithelial-to-mesenchymal transition (EMT) through various mechanisms. For example, circSTK3 upregulates EMT-associated genes; circSKA3 directly binds to and stabilizes snail family transcriptional repressor 2 (*SNAI2*), a core regulator of EMT; and circEIF3I increases non-SMC condensin I complex subunit H (*NCAPH*) expression by sequestering miR-328-3p, facilitating CRC progression [14,15,16].

Protein arginine methyltransferase 1 (PRMT1) is the predominant and most abundant type I enzyme of the human PRMT family. It catalyzes the formation of monomethylarginine and asymmetric dimethylarginine on arginine residues. This post-translational modification targets diverse protein substrates, including histones and transcription factors, regulating epigenetic programs and modulating transcription factor activity to drive carcinogenesis [17]. *PRMT1* overexpression is associated with poor prognosis in gastrointestinal, breast, and renal cancers [18,19,20,21]. In particular, splice variant 2 of *PRMT1* has been proposed as a molecular tissue biomarker of unfavorable prognosis in colon cancer [21]. Currently, PRMT1 inhibitors are under investigation as potential therapeutic targets in preclinical studies and clinical trials [22]. Recent high-throughput sequencing studies have identified multiple circ-PRMT1 transcript variants in breast cancer. Early analyses suggest that these variants may possess a regulatory role with potential prognostic implications; however, further research is needed to examine their clinical relevance in this malignancy [23,24].

In the current study, prompted by the multifaceted role of PRMT1 in epigenetic and transcriptional dysregulation, the established clinical significance of particular splice variants of this gene in various human cancers including colon cancer [21,25], and the recent discovery of circ-PRMT1 and its variants, we examined its potential clinical significance in colorectal adenocarcinoma, the most frequent type of CRC, after developing a sensitive and accurate molecular assay for its specific quantification.

## 2. Results

### 2.1. Circ-PRMT1 Expression Is Higher in Colorectal Adenocarcinoma Tissue Specimens than in Their Adjacent Non-Cancerous Tissues

The median age of colorectal adenocarcinoma patients was 67.5 years old (range: 35–93; interquartile range: 58–75). Table 1 shows the clinicοpathological features of the tumor tissues included in this study. Most of the colorectal adenocarcinoma patients have a tumor located in the colon. Among the 210 colorectal adenocarcinoma patients, over half of them had received chemotherapy after surgery, while most tumors were classified as being of histological grade II and TNM stage III.

**Figure 1:**
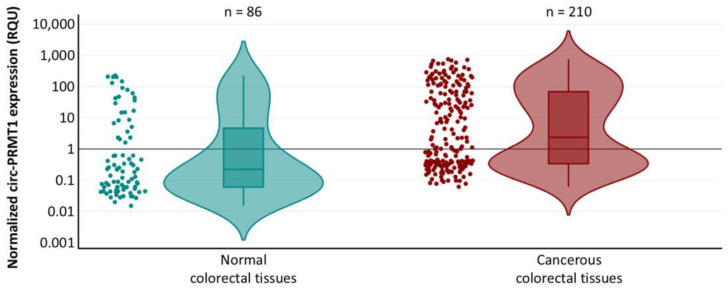
Violin plots showing the distributions of circ-PRMT1 expression levels in colorectal adenocarcinomas and non-cancerous colorectal tissues. The y-axis is on a log_10_ scale.

**Figure 2:**
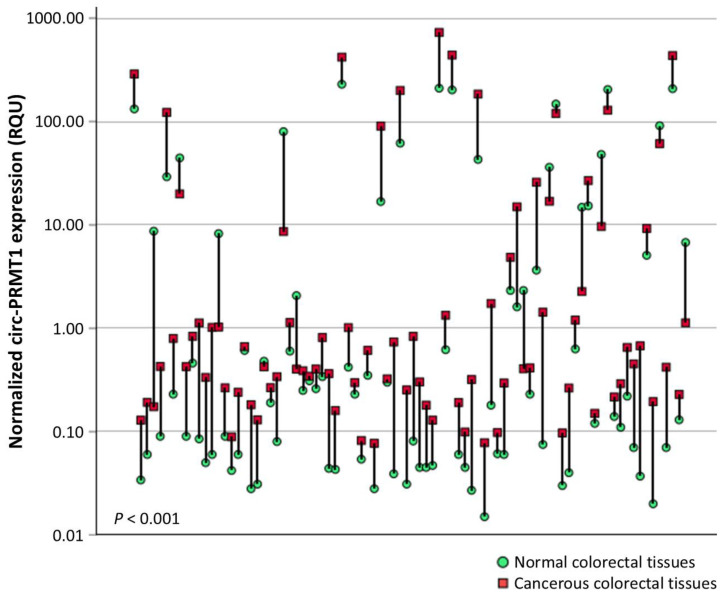
A comparison of the circ-PRMT1 expression levels in 86 cancerous colorectal tissues vs. their normal counterparts. The *p* value was estimated by the Wilcoxon signed-rank test.

Circ-PRMT1 levels in colorectal adenocarcinoma samples ranged from 0.061 to 750.48 RQU, with a median of 2.38 RQU, whereas in non-cancerous tissues the levels fluctuated from 0.015 to 229.64 RQU, with a median of 0.23 RQU (Figure 1). Comparison of circ-PRMT1 expression levels between pairs of cancerous and normal tissues showed profound upregulation (*p* < 0.001) in the former (72 out of 86 tissue pairs; 83.7%) (Figure 2). The *p* value was calculated by the Wilcoxon signed-rank test.

**Figure 3:**
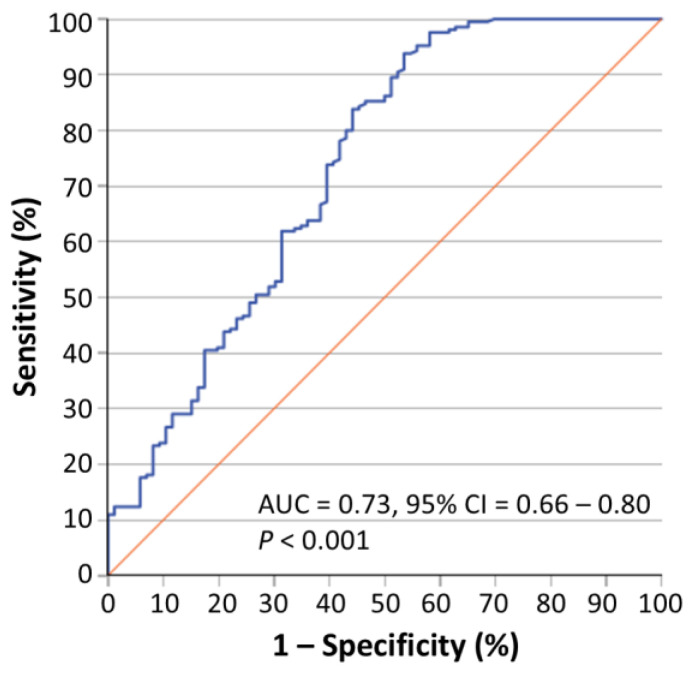
A receiver operating characteristic (ROC) curve illustrating the ability of circ-PRMT1 expression to efficiently distinguish colorectal adenocarcinoma from normal colorectal tissue. The *p* value was estimated by the Mann–Whitney *U* test. Abbreviations: AUC, area under the ROC curve; CI, confidence interval.

In accordance with these findings, receiver operating characteristic (ROC) curve analysis demonstrated that circ-PRMT1 expression may successfully distinguish colorectal adenocarcinoma from normal colorectal tissue (AUC = 0.73, 95% CI = 0.66–0.80, *p* < 0.001) (Figure 3). The *p* value was calculated by the Mann–Whitney *U* test. On the other hand, circ-PRMT1 expression did not differ significantly among tumors of patients at different TNM stages, as shown by using the Kruskal–Wallis *H* test and the Jonckheere–Terpstra test, where appropriate.

### 2.2. Circ-PRMT1 Overexpression Is an Unfavorable Prognosticator of Patients’ Outcome

Next, we examined any correlation existing between circ-PRMT1 expression and the DFS and/or OS of colorectal adenocarcinoma patients. In the DFS analysis, 172 colorectal adenocarcinoma patients that did not present distant metastasis were included. Among these, 61 patients (35.5%) were diagnosed with disease recurrence during the accrual follow-up period. For the OS analysis, 198 colorectal adenocarcinoma patients with complete available follow-up data were included, while 91 cancer-related deaths (45.6%) were recorded within this cohort.

**Figure 4:**
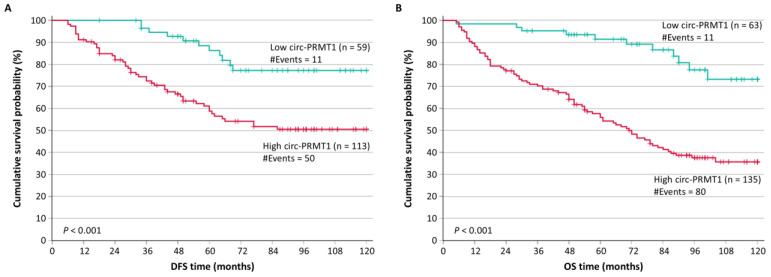
Kaplan–Meier survival curves for the disease-free survival (DFS) and overall survival (OS) of colorectal adenocarcinoma patients. Patients with neoplasms overexpressing circ-PRMT1 had significantly poorer DFS (**A**) and OS (**B**) than patients with tumors expressing low circ-PRMT1 levels. The *p* values were estimated by the Mantel–Cox (log-rank) test.

Circ-PRMT expression values were classified into two categories (high or low expressors), as described in detail in the Materials and Methods Section. Colorectal adenocarcinoma patients with circ-PRMT1 overexpression were shown to have significantly lower DFS probabilities (*p* < 0.001) compared to patients with lower circ-PRMT1 levels (Figure 4A). Additionally, the unfavorable prognostic value of circ-PRMT1 overexpression was observed in OS analysis, which revealed that colorectal adenocarcinoma patients with circ-PRMT1 overexpression had significantly poorer OS (*p* < 0.001) (Figure 4B). These results were also confirmed by univariate Cox regression analysis, estimating a hazard ratio (HR) of 3.05 (*p* < 0.001) for colorectal adenocarcinoma recurrence in patients with tumors overexpressing circ-PRMT1, as well as an HR of 4.39 (*p* < 0.001) for cancer-related death, in contrast to those with lower levels of circ-PRMT1 (Table 2).

### 2.3. Circ-PRMT1 Overexpression Retains Its Unfavorable Prognostic Significance Independently of Other Established Prognostic Factors

Multivariate Cox regression models incorporated the tumor location, histological grade, TNM stage, and type of treatment received after tumor resection (radiotherapy or chemotherapy), as presented in Table 3. The significance of the circ-PRMT1 expression status in the prognosis of the patients’ DFS was again high (HR = 3.00; *p* = 0.001), despite being combined with the status of the aforementioned clinicopathological factors. Moreover, circ-PRMT1 retained its prognostic significance for OS (HR = 3.69; *p* < 0.001), along with other clinicopathological features of the tumor.

### 2.4. Circ-PRMT1 Is an Unfavorable Prognostic Marker in Distinct Subgroups of Colorectal Adenocarcinoma Patients

**Figure 5:**
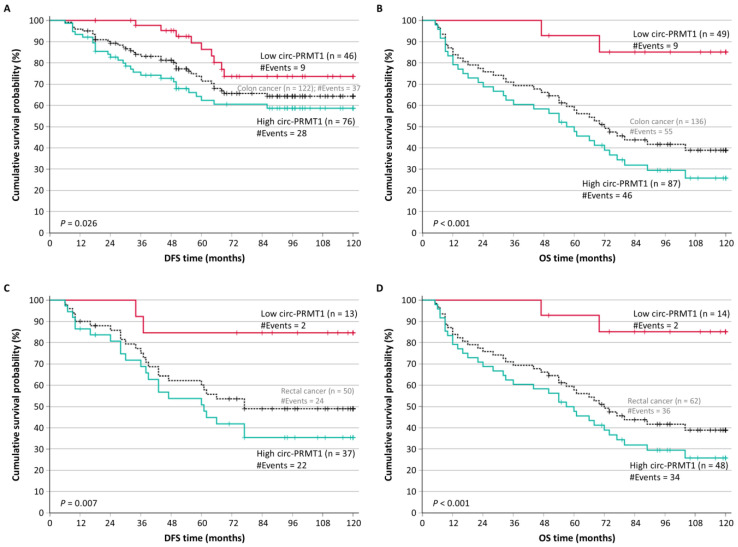
Kaplan–Meier survival curves for the disease-free survival (DFS) and overall survival (OS) of colorectal adenocarcinoma patients, stratified based on their tumor location. Patients with colon tumors that overexpress circ-PRMT1 had inferior DFS (**A**) and OS (**B**) than those with colon tumors with lower circ-PRMT1 levels. Similarly, colorectal adenocarcinoma patients with rectal tumors overexpressing circ-PRMT1 had poorer DFS (**C**) and OS (**D**) than those with rectal tumors showing lower expression of circ-PRMT1. The *p* values were estimated by the Mantel–Cox (log-rank) test.

After the stratification of colorectal adenocarcinoma patients according to their tumor location, we noticed that patients with colon tumors (122 for DFS and 136 for OS) and overexpression of circ-PRMT1 showed significantly shorter DFS intervals (*p* = 0.026) and OS intervals (*p* < 0.001) (Figure 5A,B). Similarly, as shown in Figure 5C,D, colorectal adenocarcinoma patients with rectal tumors (50 for DFS and 62 for OS) and lower levels of circ-PRMT1 had significantly higher probabilities of both DFS (*p* = 0.007) and OS (*p* < 0.001).

**Figure 6:**
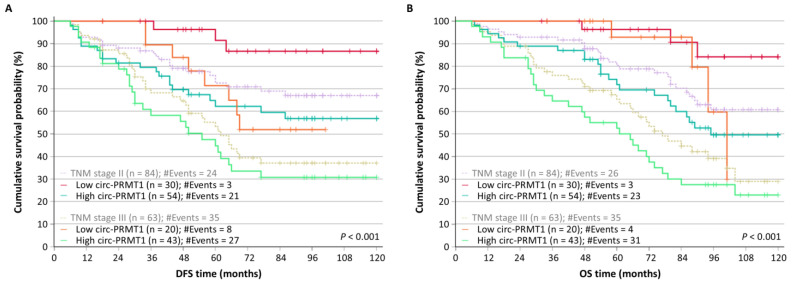
Stratified Kaplan–Meier survival curves for the disease-free survival (DFS) and overall survival (OS) of colorectal adenocarcinoma patients with TNM stage II or III. circ-PRMT1 overexpression may be used in addition to TNM staging, particularly for these two TNM stages, to distinguish those patients with higher DFS (**A**) and OS (**B**) probabilities. The *p* values were estimated by the Mantel–Cox (log-rank) test.

Furthermore, we stratified colorectal adenocarcinoma patients according to TNM stage; a total of 84 colorectal adenocarcinoma patients with complete survival data had TNM stage II disease and another 63 had TNM stage III disease. According to the Kaplan–Meier curves, patients with TNM stage II and III and high levels of circ-PRMT1 had both poorer DFS (Figure 6A) and OS (Figure 6B), in contrast to patients with the same TNM stages and low levels of circ-PRMT1 (*p* < 0.001 in both cases).

## 3. Discussion

Colorectal cancer (CRC) ranks as the third most common malignancy and the second leading cause of cancer-related mortality globally. Although it is considered a disease of older adults, CRC incidence is rising alarmingly among younger populations. Projections suggest an overall 55% increase in cases and a 65% surge in mortality by 2040, making CRC a critical public health challenge. To address its molecular complexity, research consortia like ColoMARK and DISCERN aim to elucidate mechanisms driving CRC pathogenesis. The molecular heterogeneity of localized colorectal tumors contributes to variable metastatic potential and ultimately poor prognosis for some patients. Additionally, current treatment options are usually accompanied by long-term morbidity, underscoring the need for biomarkers to guide therapeutic decisions in patients most likely to benefit. Such precision would optimize treatment efficacy while minimizing unnecessary side effects.

CircRNAs are an emerging class of RNA molecules implicated in CRC development and progression through various mechanisms, including miRNA sponging, RBP interaction, alternative splicing regulation, and epigenetic modulation. Numerous circRNAs have been found aberrantly expressed in the blood and tissues of CRC patients compared to healthy controls [26]. Their closed circular structure, lacking 5′ and 3′ ends, confers resistance to exonuclease degradation, ensuring high stability in tissues and biological fluids [27,28]. This stability, paired with distinct, cancer-specific expression patterns, makes circRNAs promising noninvasive biomarkers for CRC diagnosis and prognosis. Although their low abundance and sequence similarity to linear RNAs pose analytical challenges, advances in high-throughput and long-read sequencing technologies have improved their detection and quantification [29].

The human *PRMT1* gene is located at chromosome 19 and encodes the predominant type I protein arginine methyltransferase. It plays a critical role in embryonic development, as its complete deletion in mice leads to embryonic lethality [29]. Moreover, *PRMT1* is a key regulator of intestinal cell proliferation and differentiation [30]. Recent high-throughput studies have identified multiple alternative splice variants of *PRMT1* [23,24,31]. Several of these variants are found aberrantly expressed in CRC cell lines and tissues and are associated with the prognosis of patients [25,31].

Our study identifies circ-PRMT1 as a clinically significant predictor of colorectal adenocarcinoma progression, with overexpression detected in 83.7% of tumors compared to matched non-cancerous tissues. *PRMT1* is a well-characterized oncoprotein in CRC driving epidermal growth factor receptor (*EGFR*) activation via arginine methylation and conferring cetuximab resistance [32]. Besides direct EGFR modulation, PRMT1 exerts significant epigenetic control by methylating histone H4, which recruits the chromatin remodeler SWI/SNF-related BAF chromatin remodeling complex subunit ATPase 4 (*SMARCA4*). This interaction forms a transcriptional complex that amplifies *EGFR* expression, further increasing oncogenic signaling [33]. circ-PRMT1 exhibits distinct molecular features that may independently influence tumor development and progression. Unlike its linear counterpart, circ-PRMT1 transcript variants harbor microexons, extended exon boundaries, and putative miRNA-binding motifs, suggesting roles as a competing endogenous RNA or templates for novel protein isoforms [23]. Recent studies report that circRNAs interact with RBPs to modulate the mRNA stability of target genes and, consequently, regulate oncogenic pathways [34]. circ-PRMT1 transcript variants are predicted to interact with RNA binding motif protein 45 (RBM45), serine and arginine rich splicing factor 9 (*SRSF9*) and heterogeneous nuclear ribonucleoprotein F (*HNRNPF*) [24]. RBM45 downregulation suppresses hepatocellular carcinoma proliferation, while SRSF9 stabilizes oncogenic transcripts like DSN1, which correlates with lymph node metastasis and an advanced stage of CRC [35,36]. HNRNPF contributes to cancer progression by regulating the splicing of oncogenic transcripts, such as the macrophage-stimulating 1 receptor (*MST1R*), leading to the activation of downstream signaling pathways implicated in tumorigenesis [37]. Genetic alterations in *HNRNPF* that produce defective protein isoforms have been identified in inflammatory disorders, which are directly associated with an elevated risk of CRC [38]. These interactions render circ-PRMT1 a potential mediator of mRNA processing or translational efficiency in critical pathways, providing potential mechanistic insights into its association with advanced TNM stages and poor survival outcomes, similar to splice variant 2 of this gene in colon cancer [21].

Elevated circ-PRMT1 expression was associated with significantly shorter DFS and OS in colorectal adenocarcinoma patients. Multivariate Cox regression models confirmed that circ-PRMT1 is an independent prognostic factor for survival outcomes, irrespective of tumor location, histological grade, TNM stage, or therapy. These observations are in accordance with previous results regarding the prognostic role of *PRMT1* mRNA in CRC and consistent across patient subgroups defined by the tumor anatomical site [25]. Stratified analysis of circ-PRMT1 expression in colorectal adenocarcinoma subgroups further validated its association with adverse clinical outcomes. Elevated circ-PRMT1 levels independently predicted shorter DFS and OS in patients with localized stage II–III tumors, highlighting its utility in refining prognostic stratification within this subset. These findings address the unmet need for molecular biomarkers that can guide treatment and help to select the most beneficial therapeutic strategies, particularly in TNM stage II colorectal adenocarcinoma. In the context of ongoing controversy surrounding adjuvant chemotherapy administration for stage II patients, circ-PRMT1 may facilitate the identification of high-risk individuals who could benefit from intensified therapies while alleviating low-risk patients from unnecessary treatment-related toxicity [39]. A similar trend in circ-PRMT1 expression was observed across subgroups stratified by tumor location, with elevated circ-PRMT1 levels consistently associated with poor prognosis regardless of anatomical site.

The consistent association of circ-PRMT1 expression with unfavorable survival outcomes, regardless of TNM stage or treatment approach, increases its clinical utility as an ancillary molecular marker for refining patient risk classification. The intrinsic advantages of circRNAs, including their covalently closed structure, which ensures remarkable stability in body fluids, further expand the clinical applicability of circ-PRMT1, enabling its use for noninvasive monitoring in colorectal adenocarcinoma, as previously reported for several other circRNAs [40].

In summary, this original research study provides evidence that circ-PRMT1 overexpression represents a promising molecular biomarker of poor prognosis in colorectal adenocarcinoma, not depending on other established prognostic factors such as TNM staging. However, our study has several limitations, including its retrospective nature, single-center design, potential cohort bias, lack of validation in independent cohorts of CRC patients, and statistical shortcomings since the number of patients in particular subgroups is rather low. Such key limitations may challenge the robustness of the results of any study, making them preliminary and hypothesis-generating rather than practice-changing or clinically actionable; however, our findings pave the way for further studies in larger cohorts to thoroughly evaluate the prognostic potential of this circRNA in colorectal adenocarcinoma.

## 4. Materials and Methods

### 4.1. Sample and Clinocopathological Data Collection

The tissue sample biobank exploited in this study included 210 primary colorectal adenocarcinoma specimens from patients who had undergone surgery at the University General Hospital “Attikon” in the past. After the surgical removal of each colorectal tumor, each cancerous tissue sample was histologically evaluated by a pathologist and snap-frozen in liquid nitrogen. For 86 cases, a matched specimen of normal colorectal tissue was also available. This study was approved by the Institutional Ethics Committee of the University General Hospital “Attikon” (approval number: 31; date 29 January 2009) and was performed in accordance with the Helsinki Declaration. All colorectal adenocarcinoma patients were informed in detail about the aim of this study and consented to provide tissue samples.

The recorded clinicopathological tumor features included tumor size and location, histological grade, and TNM stage. The TNM classification included tumor invasion (T), regional lymph node status (N), and the presence or absence of distant metastases (M). Follow-up information included the cause of death for those who succumbed to their disease and tumor recurrence (or not), along with respective dates. Follow-up information was unavailable for 12 of the 210 patients, who were, therefore, excluded from the survival analysis of the current study. In total, 26 patients out of the remaining 198 with complete follow-up data had distant metastasis (M1) at the time of surgery and were, hence, excluded only from the DFS analysis.

### 4.2. Total RNA Isolation and Reverse Transcription

After tissue homogenization, total RNA was extracted using TRIzol^®^ Reagent (Ambion™, Thermo Fisher Scientific Inc., Waltham, MA, USA) and stored in a deep freezer at −80 °C. The concentration and purity of the total RNA extracts was assessed using a BioSpec-nano Micro-volume UV–Vis Spectrophotometer (Shimadju, Kyoto, Japan), while their integrity was checked by agarose gel electrophoresis. Subsequently, 2 µg of each RNA sample was subjected to reverse transcription using M-MLV reverse transcriptase (Invitrogen™, Thermo Fisher Scientific Inc., Carlsbad, CA, USA) and random hexamers (New England Biolabs Ltd., Hitchin, UK), following the manufacturers’ instructions. Reverse transcription was performed in a MiniAmp Thermal Cycler (Applied Biosystems™, Thermo Fisher Scientific Inc., Waltham, MA, USA). Total RNA from the CRC cell line DLD-1 was used as a positive control along with each batch of total RNA extracts of tissue samples; a negative control was also included in each run.

### 4.3. Primer Designing and Real-Time Quantitative Polymerase Chain Reaction (qPCR)

Divergent primers were designed for *PRMT1* circRNA, so that only circular and not linear RNAs would be amplified during the PCR and qPCR assays. Due to the low expression levels of PRMT1 circRNAs, a first-round PCR assay was conducted for the pre-amplification of these molecules and of *GAPDH* mRNA, which was used as a reference for the qPCR assay, as previously published. The reaction mixture contained 19.4 µL of nuclease-free H_2_O, 2.5 µL of 10× KAPA Taq Buffer, 0.5 µL of a dNTP mix (containing each dNTP at an initial concentration 10 mM), 1 µL of each primer (initial concentration 10 µM), 0.5 U of KAPA Taq DNA Polymerase (KAPA Biosystems Inc., Woburn, MA, USA), and 0.5 µL of cDNA template. Pre-amplification was performed in a MiniAmp Thermal Cycler (Applied Bio-systems™) and included an initial denaturation step at 95 °C for 3 min, followed by a cycling step, carried out for 15 cycles, consisting of a denaturation step at 95 °C for 30 sec, an annealing step at 60 °C for 30 sec, and an elongation step at 72 °C for 30 s. A final elongation step was carried out at 72 °C for 1 min. cDNA from total RNA extracts of DLD-1 cells was used as a positive control, along with each batch of cDNAs derived from the total RNA extracts of tissue samples; a negative control was also included in each pre-amplification. The PCR products were diluted at a ratio of 1:50 in nuclease-free H_2_O.

A real-time qPCR assay was developed (see Supplementary Materials and Methods) In brief, the reaction, which contained a KAPA SYBR FAST qPCR Master Mix (2X) Kit (KAPA Biosystems Inc.), was performed in a QuantStudio™ 5 Real-Time PCR System (Applied Biosystems™), following a standard thermal protocol for cycling and melting, as previously described [41,42]. Each qPCR reaction was performed in duplicates to assure the reproducibility of the obtained data. The expression levels of *PRMT1* circRNA were calculated using the comparative Ct (2^−∆∆Ct^) method [43]. *GAPDH* was used as an internal control gene to normalize the PCRs for the amount of RNA added to the reverse transcription reactions [42,44,45,46,47,48,49], while DLD-1 cDNA was used as a calibrator for making qPCRs from distinct runs comparable. Moreover, the pre-amplified DLD-1 cDNA accompanying each batch of samples was used for minor corrections of Ct variability resulting from the pre-amplification step. Thus, the use of appropriate controls in each reaction (i.e., reverse transcription, pre-amplification, and qPCR) ensured that no experimental bias was generated. The relative expression in each sample was determined in RQUs by calculating the ratio of *PRMT1* circRNA to *GAPDH* molecules divided by the same ratio calculated for the calibrator. All the primers that were used are presented in Table 4.

### 4.4. Biostatistics

Biostatistical analysis was conducted with IBM SPSS Statistics software (version 29) (IBM Corp., Armonk, NY, USA). The distributions of circ-PRMT1 levels in both non-cancerous and cancerous colorectal tissue samples were non-Gaussian; therefore, only non-parametric tests (Mann–Whitney *U* test, Kruskal–Wallis *H* test, and Jonckheere–Terpstra test, where appropriate) were used to assess the significance of differences in circ-PRMT1 levels observed among patients’ groups. Moreover, the Wilcoxon signed-rank test was applied to assess the significance of differences in circ-PRMT1 expression between pairs of colorectal tissues.

The X-tile algorithm was used to generate an optimal cut-off point for circ-PRMT1, as there are no established cut-off points concerning its expression in colorectal adenocarcinoma [50]. Having corrected for the use of minimum *p*-value statistics, X-tile software (version 3.6.1) yielded an optimal cut-off of 0.40 RQU, equal to the 30th percentile, with a calculated Monte Carlo *p* value of 0.05. Next, Kaplan–Meier survival analysis was performed to investigate the potential association between circ-PRMT1 expression and patient outcomes regarding disease-free survival (DFS) and overall survival (OS). The Mantel–Cox (log-rank) test was used to evaluate any differences between the resulting Kaplan–Meier curves for DFS and OS.

To further investigate the prognostic value of circ-PRMT1 levels and estimate the hazard ratio (HR) for disease recurrence and disease-related death, univariate Cox regression analysis was performed for each variable; moreover, 95% confidence intervals (CIs) were calculated for all estimated HRs, along with the respective *p* values. Additionally, multivariate Cox regression models were built after adjustment for all significant clinicopathological prognosticators. Cox regression analyses were carried out with 1000 bootstrap samples. The bootstrap bias-corrected and accelerated (BCa) method was applied to calculate bootstrap *p* values and 95% CIs for each estimated HR.

The statistical significance level was set to be lower than 0.050 (*p* < 0.050).

## 5. Conclusions

In this study, we showed the prognostic significance of circ-PRMT1 overexpression in colorectal adenocarcinoma to be an independent molecular predictor of poor DFS and OS. Our results demonstrate the clinical value of this circRNA in CRC and pave the way for further studies in larger cohorts to thoroughly evaluate its potential as a prognostic molecular biomarker in this malignancy.

## Figures and Tables

**Table 1:** The clinicopathological features of colorectal adenocarcinoma patients in the current study.

Variable	Number of Patients (%)
Gender	
Male	110 (52.4%)
Female	100 (47.6%)
**Tumor location**	
Colon	143 (68.1%)
Rectum	67 (31.9%)
**Histological grade**	
I	19 (9.0%)
II	160 (76.2%)
III	31 (14.8%)
**T (tumor invasion)**	
T1	6 (2.9%)
T2	25 (11.9%)
T3	130 (61.9%)
T4	49 (23.3%)
**N** **(nodal status)**	
N0	118 (56.2%)
N1	54 (25.7%)
N2	38 (18.1%)
**M (distant metastasis)**	
M0	184 (87.6%)
M1	26 (12.4%)
**TNM stage**	
I	28 (13.3%)
II	85 (40.5%)
III	71 (33.8%)
IV	26 (12.4%)
**Radiotherapy**	
No	166 (83.8%)
Yes	32 (16.2%)
**Chemotherapy**	
No	85 (42.9%)
Yes	113 (57.1%)

Abbreviation: TNM, tumor, node, and metastasis.

**Table 2:** Univariate Cox regression analysis results examining the prognostic potential of circ-PRMT1 expression status with regard to colorectal adenocarcinoma patients’ survival.

	Covariate	HR	95% CI	*p* Value ^1^	BCa 95% CI	Bootstrap *p* Value ^1^
**Disease-free survival (DFS)**	circ-PRMT1 expression					
	Low	1.00				
	High	3.05	1.59–5.86	*<0.001*	1.64–6.9	*0.001*
	Tumor location					
	Colon	1.00				
	Rectum	1.64	0.98–2.74	0.059	1.02–2.6	0.052
	Histological grade					
	I	1.00				
	II	1.73	0.62–4.81	0.29	0.66–2.6 × 10^4^	0.24
	III	3.06	0.97–9.61	0.056	0.81–7.0 × 10^4^	*0.035*
	TNM stage					
	I	1.00				
	II	4.37	1.03–18.49	*0.045*	1.35–2.8 × 10^4^	*0.029*
	III	9.95	2.39–41.47	*0.002*	3.21–7.6 × 10^4^	*0.001*
	Radiotherapy					
	No	1.00				
	Yes	1.78	0.99–3.19	0.052	0.97–3.04	*0.044*
	Chemotherapy					
	No	1.00				
	Yes	2.32	1.32–4.06	*0.003*	1.31–4.35	*0.004*
**Overall survival (OS)**	circ-PRMT1 expression					
	Low	1.00				
	High	4.39	2.33–8.24	*<0.001*	2.52–9.56	*0.001*
	Tumor location					
	Colon	1.00				
	Rectum	1.53	1.01–2.33	*0.047*	0.98–2.42	0.056
	Histological grade					
	I	1.00				
	II	1.32	0.61–2.88	0.48	0.6–3.9	0.51
	III	2.72	1.14–6.48	*0.024*	1.13–8.05	*0.023*
	TNM stage					
	I	1.00				
	II	2.60	0.91–7.48	0.075	0.83–3.6 × 10^4^	0.062
	III	5.76	2.03–16.29	*<0.001*	1.8–6.2 × 10^4^	*0.005*
	IV	34.85	11.71–103.72	*<0.001*	9.69–4.7 × 10^5^	*0.001*
	Radiotherapy					
	No	1.00				
	Yes	1.67	1.02–2.75	*0.042*	0.91–2.95	0.057
	Chemotherapy					
	No	1.00				
	Yes	1.78	1.15–2.77	*0.010*	1.15–2.77	*0.013*

^1^ Statistically significant *p* values are shown in italics. Abbreviations: BCa, bias-corrected and accelerated; CI, confidence interval; HR, hazard ratio.

**Table 3:** Multivariate Cox regression analysis results examining the independence of circ-PRMT1 expression status with regard to colorectal adenocarcinoma patients’ survival.

	Covariate	HR	95% CI	*p* Value ^1^	BCa 95% CI	Bootstrap *p* Value ^1^
**Disease-Free Survival (DFS)**	circ-PRMT1 expression					
	Low	1.00				
	High	3.00	1.55–5.79	*0.001*	1.59–7.6	*0.003*
	Tumor location					
	Colon	1.00				
	Rectum	2.56	1.26–5.18	*0.009*	1.11–8.23	*0.029*
	Histological grade					
	I	1.00				
	II	1.07	0.37–3.11	0.90	0.28–2.3 × 10^4^	0.87
	III	1.61	0.48–5.35	0.44	0.36–3.5 × 10^4^	0.43
	TNM stage					
	I	1.00				
	II	4.86	1.10–21.61	*0.038*	1.23–8.8 × 10^4^	*0.015*
	III	9.74	2.09–45.39	*0.004*	2.09–1.9 × 10^5^	*0.002*
	Radiotherapy					
	No	1.00				
	Yes	0.48	0.21–1.09	0.080	0.19–0.98	0.086
	Chemotherapy					
	No	1.00				
	Yes	1.37	0.72–2.63	0.34	0.61–3.03	0.38
**Overall Survival (OS)**	circ-PRMT1 expression					
	Low	1.00				
	High	3.69	1.95–6.98	*<0.001*	1.81–11.26	*0.001*
	Tumor location					
	Colon	1.00				
	Rectum	1.42	0.83–2.44	0.20	0.73–2.62	0.22
	Histological grade					
	I	1.00				
	II	0.78	0.34–1.79	0.56	0.29–2.27	0.62
	III	1.09	0.43–2.75	0.86	0.35–4.18	0.88
	TNM stage					
	I	1.00				
	II	2.97	1.00–8.80	*0.049*	0.73–5.4 × 10^4^	*0.042*
	III	6.98	2.26–21.58	*<0.001*	1.7–2.2 × 10^5^	*0.001*
	IV	36.97	11.31–120.83	*<0.001*	8.18–1.3 × 10^6^	*<0.001*
	Radiotherapy					
	No	1.00				
	Yes	0.96	0.50–1.83	0.90	0.47–1.89	0.90
	Chemotherapy					
	No	1.00				
	Yes	0.78	0.46–1.32	0.35	0.41–1.43	0.36

^1^ Statistically significant *p* values are shown in italics. Abbreviations: BCa, bias-corrected and accelerated; CI, confidence interval; HR, hazard ratio.

**Table 4:** The primers used in pre-amplification and real-time qPCR assays for the selective amplification of *PRMT1* circRNA and *GAPDH* mRNA.

Assay	Target	Sequence (5′ → 3′)	T_a_ (°C)	Amplicon Length (bp)
Pre-amplification	*PRMT1* circRNA	TGACTCCTACGCACACTTTGG	60	401
		TCTTTGGATGTCATGTCCTCAGC		
	*GAPDH* mRNA	CCACATCGCTCAGACACCAT	60	223
		TGACAAGCTTCCCGTTCTCA		
Real-time qPCR	*PRMT1* circRNA	AGGTGGAGAGGTGCGG	60	96
		TTGGGCTTCTCACTGCTTTCC		
	*GAPDH* mRNA	ATGGGGAAGGTGAAGGTCG	60	107
		GGGTCATTGATGGCAACAATATC		

Abbreviations: bp, base pairs; T_a_, annealing temperature.

## Data Availability

The data presented in this study are available on reasonable request from the corresponding authors.

## Supplementary Materials

The following supporting information can be downloaded at https://www.mdpi.com/article/10.3390/ijms26146683/s1.
