# Supplementary material for: Overexpression of Circular PRMT1 Transcripts in Colorectal Adenocarcinoma Predicts Recurrence and Poor Overall Survival
Source: Int J Mol Sci. 2025 Jul 11;26(14):6683. doi: 10.3390/ijms26146683 (PMC12294339; doi:10.3390/ijms26146683)
Supplement: Supplementary file 1 [file ijms-26-06683-s001.zip › ijms-3715159-supplementary.pdf]

## Supplementary Materials and Methods

*Development of a nested real-time qPCR assay for the relative quantification of circ-PRMT1 expression*

The circ-PRMT1 amplicon spanning the back-splice junction of circ-PRMT1 was validated using Sanger sequencing. Then, a nested real-time qPCR assay, preceded by a pre-amplification step, was designed and optimized for the accurate quantification of circ-PRMT1 in samples of low total RNA mass. For this purpose, we performed standardization experiments with regard to cDNA input, primer concentration, and annealing temperature, $MgCl_2$ concentration, number of thermal cycles during the $1^{st}$ PCR, and dilution of the pre-amplified template of the nested real-time qPCR. Next, triplicate reactions of serial dilutions of the DLD-1 cDNA were used to generate specific melt curves for circ-PRMT1 and *GAPDH* amplicons, to validate the qPCR efficiencies. The unique melt curve of each amplicon served as evidence of the specificity of the assay, and the mean $C_t$ values were plotted against the $\log_{10}$ of DLD-1 cDNA quantity.
